# Tissue phase mapping using breath-hold 4D PCMR

**DOI:** 10.1186/1532-429X-16-S1-W30

**Published:** 2014-01-16

**Authors:** Jennifer A Steeden, Grzegorz T Kowalik, Andrew Taylor, Vivek Muthurangu

**Affiliations:** 1 UCL Centre for Cardiovascular Imaging, London, UK

## Background

Conventionally Tissue Phase Mapping (TPM) is carried out in one 2D slice. 4D PCMR has not previously been used for TPM due to long acquisition times. The purpose of this study was to develop a highly accelerated 4D PCMR sequence to acquire TPM data across the entire left ventricle (LV), during a breath-hold.

## Methods

An undersampled, prospectively triggered, stack-of-spirals, 4D PCMR sequence was developed, and reconstructed using SENSE-UNFOLD. A uniform-density spiral trajectory was used in kx-ky with 8 interleaves required to fill k-space, with acceleration of R = 4 (resulting in 2 spiral interleaves acquired per kz position). In kz, 16 slices were used, with acceleration of R = 2, resulting in 9 kz positions being acquired for each cardiac phase (calculated as 16÷2 plus one to ensure that the middle kz position is always acquired). The other sequence parameters were; TE/TR:4.0/14.4 ms, matrix size:160 × 160, FOV:500 × 500 mm, slices:16, slice thickness:8 mm, venc(x/y/z):30/30/30 cm/s. This resulted in a temporal resolution of 57.5 ms, a spatial resolution of 3.1 × 3.1 × 8 mm, and a scan time of 16 heartbeats (breath-hold), plus 7.3 s for acquisition of fully sampled coil sensitivity maps with 4NSAs (free breathing, at the end of the TPM acquisition). This 4D PCMR TPM data was acquired in 5 healthy volunteers (4M:1F, mean age:38.6 ± 6.6 yrs), and 1 patient with mild LV dyssynchrony (F, 43.3 yrs). Data Analysis: Bulk motion correction was performed, before transformation of the velocities to an internal polar coordinate system allowing motion to be described in terms of contraction (using radial velocities; Vr) and shortening (using longitudinal velocities; Vz). Peak velocities in the S (systolic) and E (early diastolic) waves were measured for the longitudinal and radial velocities within the entire myocardium, at a basal, mid and apical slice.

## Results

**Figure 1:**
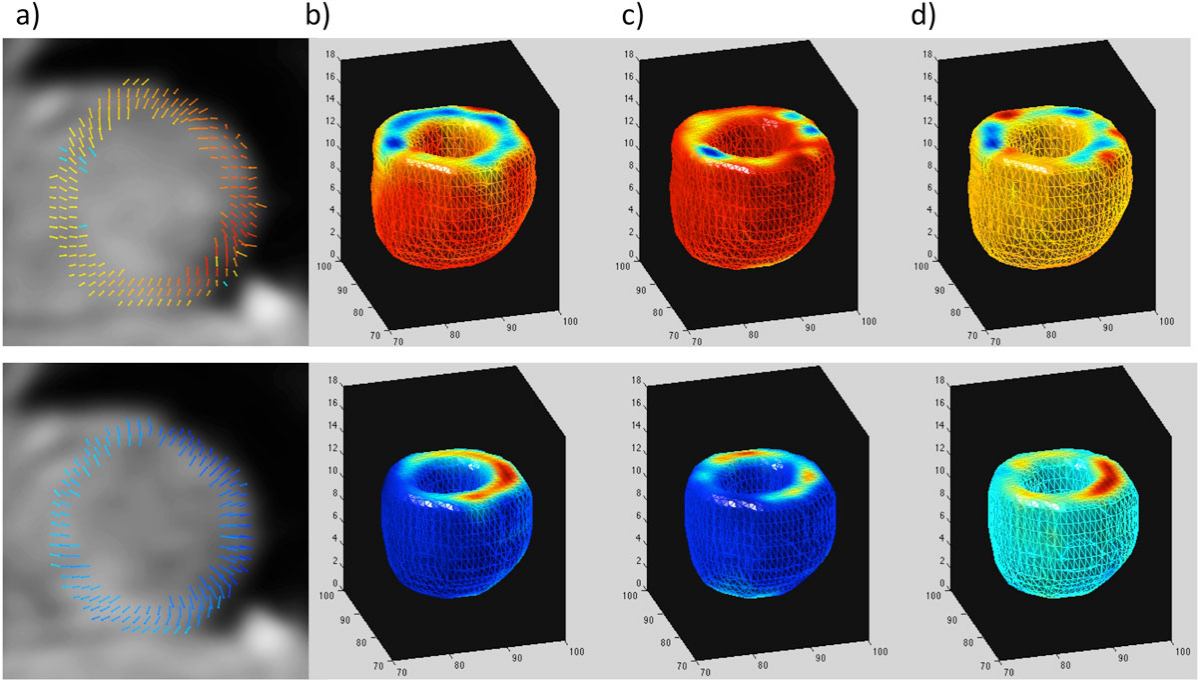
**Example data from a healthy volunteer**. Top; peak S wave, bottom; peak E wave. a) 2D vector plots from the mid slice, b) Longitudinal velocity, c) Radial velocity, d) Tangential velocity. The colors represent the velocities, with reds representing shortening/contraction/clockwise rotation, and blues representing lengthening/expansion/counter-clockwise rotation.

**Figure 2:**
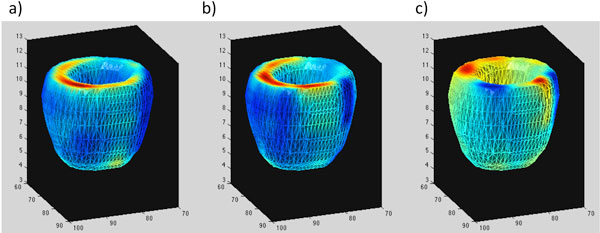
**Data from the patient showing an area of the septum with a subtle dyssynchrony during the E wave**. a) Longitudinal velocity, b) Radial velocity, c) Tangential velocity.

Data was successfully acquired and analyzed in all subjects. Figure 1 shows the calculated velocity data in one subject. In the volunteers quantitatively, the longitudinal velocities in the base/mid/apical slices were 4.9 ± 1.2/3.5 ± 1.3/2.4 ± 0.6 cm/s at the S wave, and -6.2 ± 1.3/-3.9 ± 1.4/-2.43 ± 1.5 cm/s at the E wave. The radial velocities in the base/mid/apical slices were 2.2 ± 0.4/2.7 ± 0.2/2.1 ± 0.2 cm/s at the S wave, and -2.5 ± 0.4/-3.1 ± 0.5/-2.9 ± 0.1 cm/s at the E wave. In the patient the longitudinal velocities in the base/mid/apical slices were significantly lower at the S wave (1.1/1.0/1.4 cm/s). Furthermore, there was evidence of dyssynchrony in early LV diastole as shown in Figure 2.

## Conclusions

We have shown that using a novel spiral UNFOLD SENSE PCMR sequence it is possible to obtain 4D TPM data in a breath hold. This sequence may have significant value in the assessment of LV dyssynchrony, particularly in relation to resynchronization therapy.

